# Self-regulation of charged defect compensation and formation energy pinning in semiconductors

**DOI:** 10.1038/srep16977

**Published:** 2015-11-20

**Authors:** Ji-Hui Yang, Wan-Jian Yin, Ji-Sang Park, Su-Huai Wei

**Affiliations:** 1 National Renewable Energy Laboratory, Golden, CO 80401, USA; 2 Beijing Computational Science Research Center, Beijing, 100094, China

## Abstract

Current theoretical analyses of defect properties without solving the detailed balance equations often estimate Fermi-level pinning position by omitting free carriers and assume defect concentrations can be always tuned by atomic chemical potentials. This could be misleading in some circumstance. Here we clarify that: (1) Because the Fermi-level pinning is determined not only by defect states but also by free carriers from band-edge states, band-edge states should be treated explicitly in the same footing as the defect states in practice; (2) defect formation energy, thus defect density, could be pinned and independent on atomic chemical potentials due to the entanglement of atomic chemical potentials and Fermi energy, in contrast to the usual expectation that defect formation energy can always be tuned by varying the atomic chemical potentials; and (3) the charged defect compensation behavior, i.e., most of donors are compensated by acceptors or vice versa, is self-regulated when defect formation energies are pinned. The last two phenomena are more dominant in wide-gap semiconductors or when the defect formation energies are small. Using NaCl and CH_3_NH_3_PbI_3_ as examples, we illustrate these unexpected behaviors. Our analysis thus provides new insights that enrich the understanding of the defect physics in semiconductors and insulators.

Defects often play an important role in determining semiconductor properties. As a result, in the past decades, defect analysis and control have been active research fields and powerful tools in understanding and designing material properties. Two of the most important issues in defect physics are how Fermi-level pinning and the defect formation energy vary as a function of atomic chemical potentials (i.e., growth conditions). It is well known that for a defect 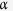 at charge state *q* in a semiconductor, defect formation energy can be written formally as a function of atomic chemical potentials 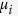 and electronic chemical potential or Fermi energy 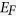 as:


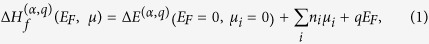


where 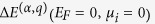 is the formation energy when the Fermi energy level is at the valence band maximum (VBM) (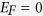) and the atomic chemical potentials of the elements 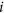 have the energies of the elements in the bulk form 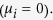 This formula and associated defect formation energy vs Fermi energy plot are commonly used to analyze defect properties of semiconductors^1^,^2^,^3^,^4^,^5^,^6^,^7^,^8^,^9^,^10^,^11^,^12^, and in many analyses, two assumptions are implicitly made. First, defect formation energies 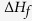 can be individually tuned by changing atomic chemical potentials and/or Fermi energy levels. Second, Fermi levels will be pinned at the lowest crossing point of acceptor and donor formation energy vs. Fermi energy lines at given atomic chemical potentials, as shown in Fig. 1. These two assumptions are often used as theoretical guidance for experiments to tune material properties by controlling growth conditions.

However, in the above rough analyses, two important facts are omitted. First, free carriers from thermal band-edge excitations will always be present at finite temperatures and will affect carrier densities and Fermi-level positions. As a result, there will be couplings between defect states and free carriers induced by band-edge states—especially when thermal band-edge excitations are strong (i.e., the effective densities of band-edge states are comparable to or even larger than the defect density of states). However, the effects of this coupling and free carriers on defect properties and Fermi-level pinning in semiconductors are not reflected in plots like Fig. 1. Second, in general, chemical potentials and Fermi levels are not independent variables and they are often entangled together. As a result, defect formation energy dependence on the atomic chemical potentials and/or Fermi level cannot be independently varied to tune defect densities. Therefore, the effects of the entanglement should be carefully examined and better understood.

To accurately determine the equilibrium Fermi level positions in a semiconductor at a finite temperature, standard procedures require solving the detailed balance equations numerically. Given the formation energies of all the defects in Eq. (1), the defect densities of 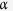 at charge state 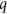 can be obtained by:


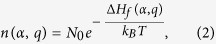


where 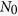 is the total density of possible sites that can form 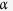 at charge state 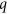, 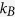 is Boltzmann constant, and T is temperature. Besides the defect densities, the thermally excited electron density 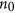 and hole density 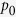 are also needed to be taken into consideration, which are:


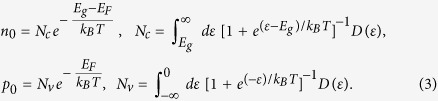


Here, 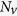 (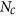) is the effective density of states (DOS) of the valence band edge (conduction band edge) that can donate electrons to (accept electrons from) the Fermi reservoir, 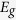 is the band gap, and 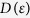 is the electron density of state with zero energy set at VBM. Under parabolic approximations, 
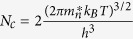
 and 
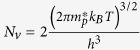
, in which 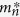 and 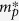 are DOS effective masses of electrons and holes, respectively, taking into account spin degeneracy and spin-orbital coupling. Finally, the charge neutralization condition in a semiconductor system requires:


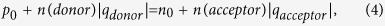


where the sum over all the donors and acceptors is implicitly indicated. By solving Eqs (1 self-consistently, one can get the balanced Fermi level positions, defect densities, and free carrier densities under equilibrium conditions given a finite temperature and certain atomic chemical potentials. In the commonly used defect formation energy vs Fermi energy plot like Fig. 1, the effects of free carriers as described in Eq. (4) are not explicitly included. Consequently, using Fig. 1 to estimate the Fermi-level pinning position can sometimes be misleading, especially when 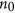 or 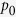 is large compared to defect densities. Besides, while Eq. (1) indicate that defect concentrations can be tuned independently by atomic chemical potentials, in reality, the atomic chemical potentials and Fermi levels are entangled: once the atomic chemical potentials change, the defect formation energy and defect concentration would change; thus, according to Eq. (4) the Fermi level will change, which in turn will lead to the additional change of defect formation energy and defect concentration. How the entanglement affects defect behaviors is also not clearly understood from just Fig. 1.

In this paper, using first-principles calculation methods combined with thermodynamic analysis, we proposed a corrected and a more general physical picture to determine Fermi levels more accurately than Fig. 1 by taking into account of free carriers induced band-edge state excitations. Based on our picture, we clarify that: (1) because the pinning position of Fermi-level is determined not only by defect states but also by free carriers from thermally excited band-edge states. The thermally excited band-edge states should be treated in the same footing as the defect states, where the valence band states should be treated as effective donors and the conduction band states should be treated as effective acceptors; (2) when thermal band-edge excitations are weak (e.g., in wide-gap ionic materials) or the defects can form easily (e.g., in some multinary compounds), which can be easily judged from our picture, charge-compensated defect formation will be self-regulated (i.e., donors will be always accompanied by acceptors); and (3) surprisingly, when the self-regulation mechanism kicks in, the defect formation energies will be independent to the changes of atomic chemical potentials, thus the defect formation energy and the defect density will be pinned and cannot be tuned by changing the atomic chemical potentials of the host elements. We demonstrate our picture by applying our analysis to the prototype ionic system NaCl to explain why this self-regulation behavior can easily happen in systems with large bandgaps. We also show that this interesting self-regulation behavior can also occur in some special small bandgap systems such as CH_3_NH_3_PbI_3_ (denoted MAPbI_3_ hereafter), which is an emerging material currently under intensive study due to its unique material properties for solar cell applications, because for MAPbI_3_, the defect forming energies are very low despite its relatively small bandgap, so Schottky defects are abundant. The formation energies of these Schottky defects will not change with the atomic chemical potential of MA or I once the atomic chemical potentials of the compound MAI is given (similarly for PbI_2_). Our analysis and insights in these fundamental issues thus provide better understanding of defect physics in these important semiconducting materials.

## Results

We start from general thermodynamic analysis to show how Fermi-level will be pinned in the presence of free carriers induced from thermal band-edge excitations and defect excitations, and how defects can be self-regulated to form in a charge-compensated manner. We use a prototype ionic system 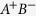 as an example. For simplicity, we consider vacancies as the only dominant defects and assume they are all at their ionized states. Note that, in real systems, the dominant defects can be in any defect formats (i.e. interstitials, antisites, complexes, etc.). No matter what the defects are, our conclusions still holds and are not only limited for Schottky defects. Extension to other ionic or not-so-ionic systems and other type of defects should be straightforward.

In common defect analysis procedures, the defect formation energies are functions of the chemical potentials and Fermi levels as follows:


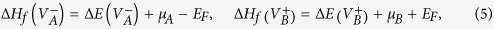


where 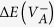 or 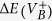 is the formation energy at 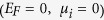. The stability of the host requires that 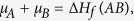 which is the formation energy of the pure *AB* compound. Under thermodynamic equilibrium growth conditions and within the dilute limit, the densities of defect 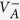 and 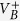 can be calculated as:


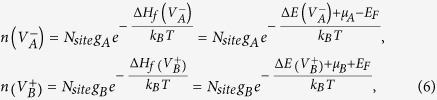


where 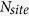 is the number of possible sites per volume for defects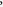 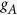 and 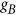 are the degeneracy factor related to possible structural configurations and electron occupations. Comparing Eqs (3) and (6), we can rewrite Eq. (3) as:


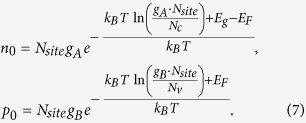


**Figure 1:**
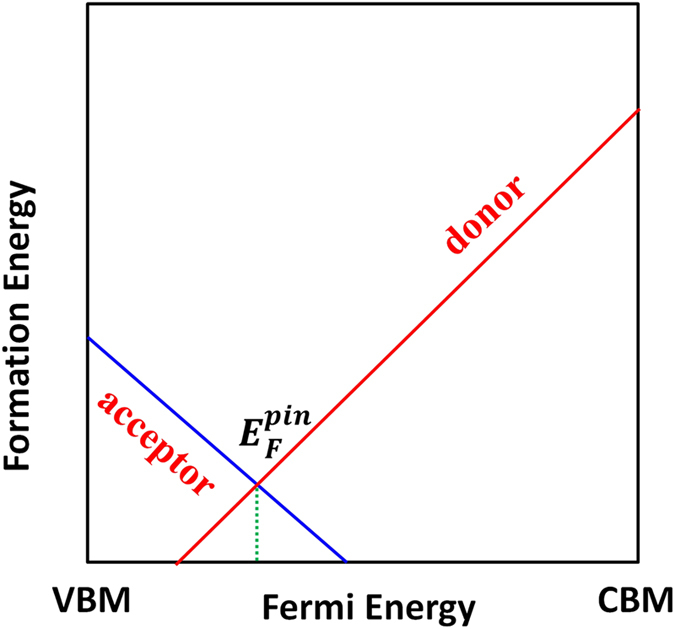
Schematic diagram to show defect formation energies as a function of Fermi energy and Fermi-level pinning at a given chemical potential in traditional defect analysis.

Comparing Eqs (6) and (7), it is clear that electron occupation at the conduction band can be treated as having a singly charged “acceptor” with its formation energy of 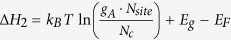 and a transition energy level at the VBM. Similarly, hole occupation in the valence band can be treated as an effective, singly ionized “donor” with its formation energy of 
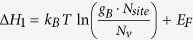
 and a transition energy level at the conduction band minimum (CBM). Note that 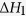 and 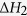 do not have an explicit dependence on atomic chemical potentials. Traditional defect analysis often omits these two “defects” when analyzing the defect formation energies as functions of Fermi levels under given chemical potentials (see Fig. 1).

**Figure 2:**
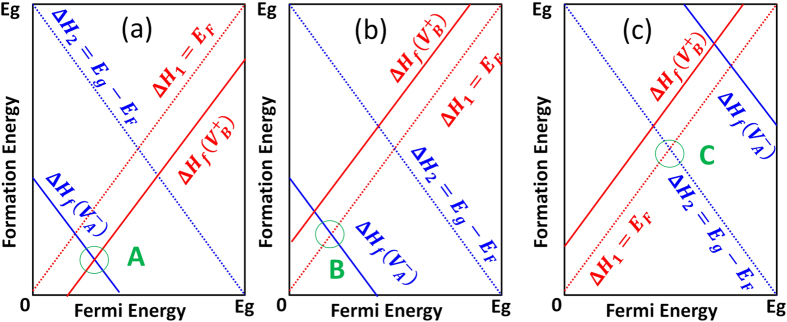
Diagrams to show different cases of Fermi-level pinning, taking into account band-edge “defects” at T = 0. (**a**) Type-I: both donors and acceptors are dominant over band-edge “defects.” (**b**) Type-II: only acceptors (or donors) are dominant over band-edge “acceptors” (or “donors”). (**c**) Band-edge “defects” are dominant. Note that in real systems, the dominant defects can be any defects beyond vacancies and only the relative positions of defect formation energy lines, 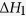 and 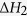 determines which scenario should be applied.

After taking into account these two “defects,” we should adopt the new pictures as shown in Fig. 2. Accordingly, three different cases can be possible. For Type-I case in Fig. 2a, we will have formation energy of 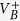 (red line in Fig. 2) lies under 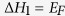 (red dashed line in Fig. 2) and the formation energy of 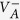 (blue line in Fig. 2) lies under 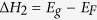 (blue dashed line in Fig. 2). Note that, at finite temperatures, 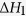 and 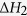 should be moved upward by 
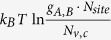
. In this case, the band-edge “defects” are not dominant and the Fermi level will be pinned at nearly the exact point A in Fig. 2a, which is the cross point of acceptor and donor formation energy lines. The deviation from point A is about 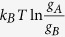, which is usually much less than 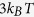.

For Type-II case (Fig. 2b), only one type of defects is dominant over band-edge “defects.” For example, in Fig. 2b, only formation energy of 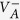 lies under 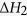 but formation energy of 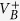 lies above 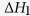; the Fermi level will be pinned at point B, which is the cross point of the acceptor formation energy line and the band-edge “donor” line 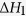. Similar things apply when only donor formation energy of 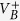 lies under 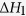 but acceptor formation energy of 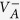 lies above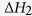. For Type-III case in Fig. 2c, band-edge “defects” are dominant over other defects and as a result, Fermi level will always be pinned near the middle of the band gap. Through the above analysis, we can see that the traditional defect analysis, which just considers defect formation energy lines, is not suitable for cases in Fig. 2b,c. Band-edge “defects” have to be considered in these two cases. One also needs to note that, if the temperature is very high, the upshift of 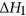 and 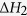, which is 
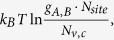
 can be large enough to change Type-II and Type-III cases to Type-I case.

Now that we've clarified the Fermi-level pinning problem, let’s focus on the Type-I case in Fig. 2a, because it can lead to some unexpected defect properties, which are contrast to and beyond traditional defect analyses. In this case, band-edge “defects” do not play important roles and the Fermi level will be pinned at point A. This is because if the 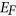 is above the A point, more 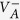 will form than 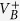 because 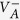 has lower formation energy at high Fermi energy, thus pulling the Fermi level back. The opposite is true if the 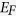 is below the A point. The result is that at the A point, the formation energy of 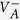 and 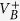 is the same, so the formation of 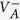 will always be accompanied by the formation of 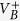 in almost equal amounts, that’s to say, most acceptors will be compensated by donors or vice versa and the formation of charge-compensated defects will spontaneously occur. Therefore, under conditions in Fig. 2a, the charged defect compensation behavior is self-regulated. Due to this defect behavior, an important consequence is the unexpected independence of the defect formation energy on the atomic chemical potentials. Note that 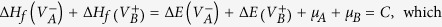 is a constant and independent on the chemical potentials and Fermi levels. This is because in this model case, the two vacancies have the same charge amount and 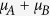 is the formation energy of the host, which is a constant. When donor and acceptor defects compensate each other as in Fig. 2a, we will have 
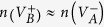
 so 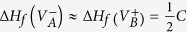 if 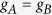. This means that both 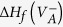 and 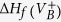 are nearly equal constants and independent on the chemical potentials and Fermi levels, which is not explicit from Eq. (1) or (2). In this case, attempts to tune defect formations by tuning chemical potentials will fail because its effect is exactly cancelled by the change of the Fermi energy. Similar defect behaviors can be generalized to binary compounds when acceptors and donors have different charge states. In this case, the defect formation energies are no longer the same but they will be both pinned and independent on chemical potentials and Fermi levels. The ratio of defect densities will always be fixed. For more complicated systems like ternary compounds, this kind of defect behavior also holds if the chemical potentials of binaries components are fixed, like CuInSe_2_ and the following discussed MAPbI_3_. Here we want to point out that the formation energy pinning is mainly applied to intrinsic dominant defects. For extrinsic impurities, only Fermi level pinning in Fig. 2 is applied. Another important consequence due to charged defect compensation is that the carrier densities could be low and make the material intrinsic because of the strong compensation of donors and acceptors; or if the absolute value of 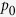 or 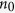 is significantly large enough, then the densities of charge-compensated intrinsic defects will be even larger, which often means the stability of the material will be affected due to formations of too many defects.

Through the above analysis, we can conclude that Fig. 2 can be regarded as a criterion of whether thermal band-edge excitations or band-edge “defects” can be neglected and whether charge-compensated defect behavior will be dominant. Note that in ionic systems, Fig. 2a can be easily satisfied because bandgaps are usually very large and defect formation energies are usually relatively small; therefore, 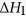 and 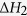 lines can lie far above the defect formation energy lines of intrinsic defects. However, in small bandgap systems, it can also be satisfied if defect formation energies are very small so their formation energy lines lie below 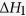 and 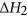. The difficulty lies that usually in small bandgap systems, it is difficult to find a chemical potential region where the acceptor formation energy is lower than 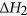 and, simultaneously, the donor formation energy is lower than 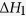. In the following, we will demonstrate our concept by applying the analysis to the wide bandgap NaCl system and narrow bandgap MAPbI_3_ perovskite solar cells.

## Discussion

### Wide bandgap NaCl system

**Figure 3:**
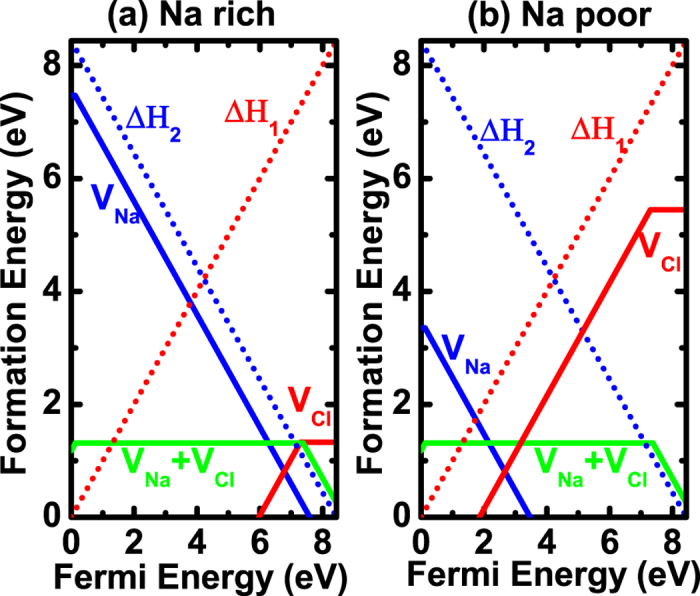
Calculated formation energies of vacancy defect as well as the band-edge “defects” as functions of Fermi levels in NaCl under (**a**) Na-rich and (**b**) Na-poor conditions.

**Figure 4:**
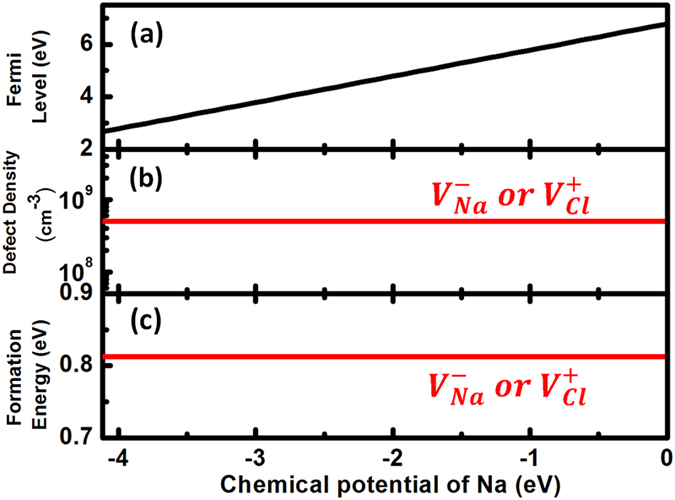
Thermodynamic simulation results in NaCl at T = 300 K. (**a**) Fermi levels, (**b**) defect density, and (**c**) defect formation energy dependence on Na chemical potentials.

As a typical ionic material, NaCl has a calculated bandgap of 8.44 eV (HSE06 calculation with exchange parameter 0.6), in agreement with the experimental value of 8.5 eV^13^. Figure 3 shows the calculated intrinsic defect properties of NaCl using 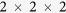 cubic supercells and 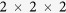 *k*-point meshes under Na-rich and Na-poor conditions, respectively, with the chemical potentials of Na and Cl, referenced to Na solid and 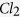 molecule, satisfying 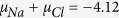 eV, which is the formation energy of NaCl compared to the experimental value of −4.26 eV^14^. Clearly, due to the very large bandgap, which can be considered as the formation energy of “neutral band-edge defects,” the formation energy of negatively charged Na vacancy is always lower than 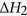 and the formation energy of positively charged Cl vacancy is always lower than 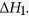 According to Fig. 2a, we can expect: (1) Na vacancy and Cl vacancy will always compensate each other in nearly equal amounts; (2) the formation energies of 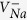 and 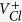 are equal and independent on the chemical potentials and Fermi levels. To confirm our expectations, we performed the standard defect analysis procedures and get the balanced Fermi levels, defect formation energies, and defect densities as functions of the chemical potentials. Our simulated results for NaCl are shown in Fig. 4. The calculated formation energy in the whole chemical potential range (Fig. 4c) is always 0.81 eV, in agreement with the experimental value of 0.75 eV^15^. The independence of formation energies of 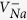 and 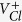 on chemical potentials and Fermi levels results from the almost exact cancelation of the variance of chemical potentials and Fermi levels, as shown in Fig. 4a. We also note that the formation energy of the neutral-bounded defect pair 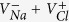 is always larger than the formation energy of isolated 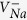 or 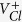 (see Fig. 3), indicating its amount is less than those of 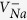 and 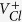 even though it has a binding energy of about 0.44 eV. As a result, the charge compensation between 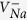 and 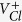 is not determined by their binding but is a result of the self-regulation in NaCl system.

### Narrow bandgap MAPbI_3_ system

As an emerging photovoltaic material, perovskite MAPbI_3_ has attracted great interest recently due to its unique material properties and high solar cell power conversion efficiency^16^,^17^,^18^,^19^,^20^,^21^,^22^,^23^,^24^. Although the defect properties of MAPbI_3_ have been extensively studied, showing the ease of defect formation to support both p-type and n-type doping by tuning chemical potentials^25^, the carrier densities are generally low and high-efficiency solar cells usually contain an intrinsic perovskite layer^26^,^27^. Theoretically, the low carrier densities are attributed to the formation of Schottky defects based on stoichiometry assumptions, which can have many effects on the material properties^27^. Here, by applying our model shown in Fig. 2, we prove that the stoichiometry assumption is naturally satisfied and the formation of full/partial Schottky defects is self-regulated in this system.

**Figure 5:**
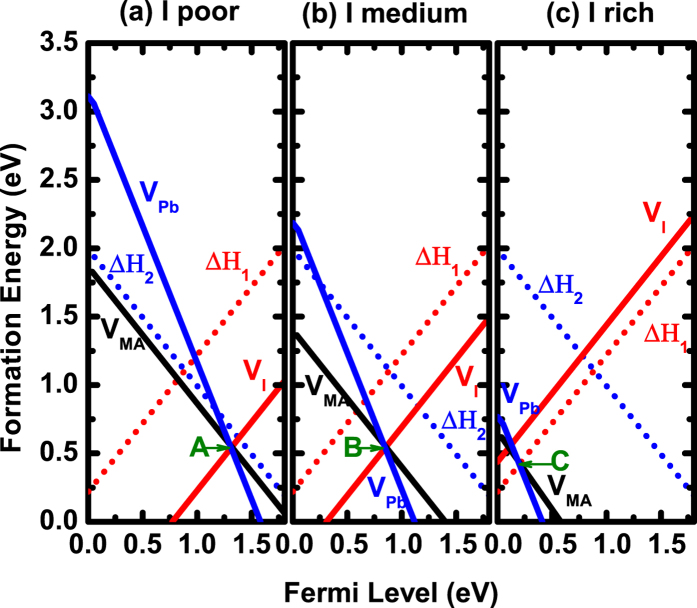
Calculated vacancy defect and band-edge “defects” formation energies as functions of Fermi levels for MAPbI_3_ at three chemical potential conditions at T = 300 K. (**a**) 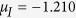 eV,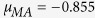 eV, and 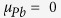
eV. (**b**) 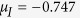
eV, 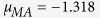
eV, and 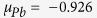
eV. (**c**) 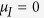
eV,
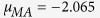
eV,
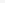
and
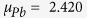
eV. The defect calculations are performed using 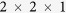 supercells and 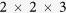 *k*-point meshes.

**Figure 6:**
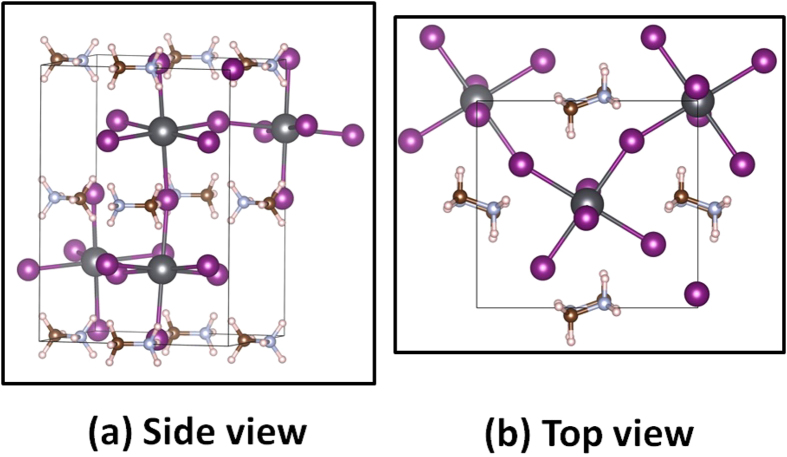
Structure of orthorhombic MAPbI_3_. (**a**) Side view and (**b**) top view.

Different from NaCl, MAPbI_3_ is a relatively small bandgap system with a calculated bandgap of 1.80 eV based on PBE calculations because the standard DFT underestimation of bandgap is somehow cancelled by the overestimation due to the omission of spin-orbit coupling^28^. Since all the dominant defects are shallow, defect properties are not expected to change with bandgap corrections. Recent HSE06 calculations by M. H. Du also show that all the dominant defects are still shallow^29^. Figure 5 shows our calculated vacancy formation energies as functions of Fermi levels under three different chemical potentials, where chemical potentials of MA, Pb, and I are referenced to CH_3_NH_2_ and H_2_ molecules, Pb solid, and I_2_ molecule, respectively. The stability of MAPbI_3_ requires 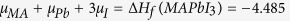
eV, 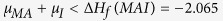
eV,and
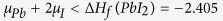
eV, where MAPbI_3_ adopts an orthorhombic structure (see Fig. 6) with organic molecules oriented either along [110] or [−110] directions^28^ and anti-ferroelectrically aligned between layers along c axis, MAI adopts a quasi-cubic NaCl structure with 4 formula in a cell, and PbI_2_ adopts a layered hexagonal structure. The calculated lattice constants of MAPbI_3_ are a = 9.26 Å, b = 8.65 Å, and c = 12.88 Å, respectively, in agreement with previous calculations^30^. Note that the cubic phase and tetragonal phase of MAPbI_3_ are not stable according to our calculations at zero temperature. Besides, because we referenced the chemical potentials to the more stable structure of MAI, we found the most stable donor defect is iodine vacancy instead of MA interstitials in Ref. 25. In either way or even if the dominant donor is MA interstitials, our concept about Fermi level and formation energy pinning still holds. The stable chemical region is so narrow (e.g., 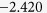
eV
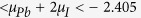
eV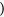 that all the chemical potentials are almost locked with 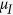. As a result, the following conditions are used in the simulations: 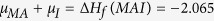 eV and 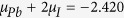 eV, corresponding to MAI-rich and PbI_2_-poor conditions.

As can be seen in Figs. 5a,b, the acceptor formation energies are lower than band-edge “acceptor” 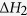 and the donor formation energy is lower than band-edge “donor” 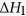. (Note that 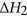 has been moved upward by 0.19 eV and 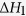 has been moved upward by about 0.22 eV at T = 300 K, according to 
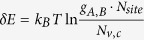
), indicating that (1) most of the intrinsic donors V_I_ and acceptors V_MA_ and V_Pb_ should be highly compensated, (2) Schottky defects are dominant and self-regulated to form, and (3) Fermi levels will be pinned close to point A in Fig. 5a or point B in Fig. 5b. However, things are different in Fig. 5c, where donor formation energy is higher than band-edge “donor” 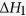. As a result, the Fermi-level position is determined not by the crossing point of donor and acceptors which is about 0.10 eV above VBM, but rather by the crossing point (point C in Fig. 5c) of band-edge “donor” 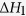 and acceptors, which is about 0.18 eV at T = 300 K.

**Figure 7:**
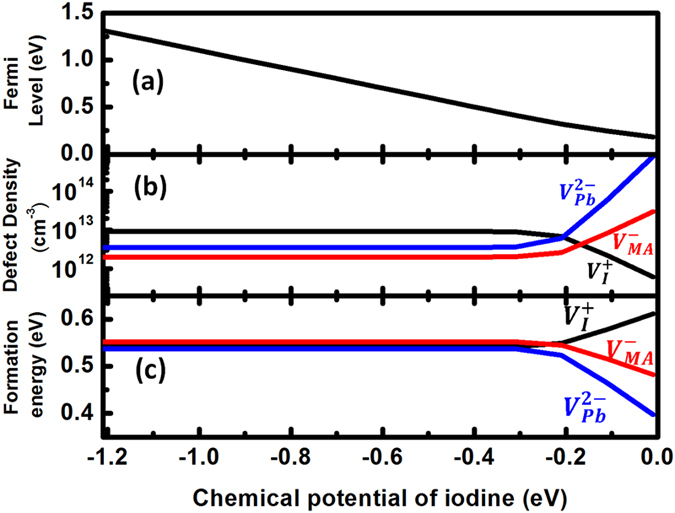
Thermodynamic simulation results in MAPbI_3_ at T = 300 K. (**a**) Fermi levels, (**b**) defect density, and (c) defect formation energy dependence on I chemical potentials.

To confirm our expectations, thermodynamic simulations are performed under MAI-rich conditions, 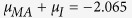
eV, and the results are shown in Fig. 7. 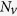 and 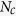 are 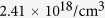 and 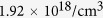 at T = 300 K, respectively, with the hole effective mass of 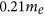 and electron effective mass of 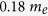
31. As can be seen in Fig. 7b, the densities of 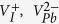, and 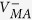 do not change with chemical potentials of iodine before 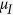 reaches −0.3 eV. Correspondingly, their formation energies are fixed as 0.542 eV, 0.538 eV and 0.552 eV, respectively, under MAI-rich and PbI_2_-poor conditions. The independence of formation energies on chemical potentials result from that Fermi levels vary linearly with and cancel almost exactly the change of the chemical potentials, as can be seen in Fig. 7a. Also, the ratios of defect densities of 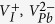 and 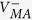 are 9.13:3.56:2.01, meaning that these three defects are always formed in partial Schottky forms with the deficiency of PbI_2_ larger than the deficiency of MAI. Similarly, we can expect the deficiency of MAI to be larger than that of PbI_2_ under MAI-poor and PbI_2_-rich conditions. Indeed, our simulations show that the ratios of defect densities of 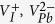, and 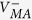 are 8.49:2.33:3.83 under MAI-poor and PbI_2_-rich conditions, corresponding to their formation energies of 0.544  V, 0.549 eV, and 0.536 eV, respectively. Because Schottky defects are dominant, the carrier concentrations are very low (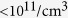) when 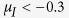
eV and MAPbI_3_ is expected to be intrinsic, which explains the popularity of 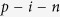 based devices. Besides, Schottky defects might be related to the stability of MAPbI_3_ as ions can easily diffuse through vacancies. Only in a relatively small chemical potential region (e.g., −0.3 eV
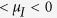
eV), can MAPbI_3_ be slightly p-type when acceptor densities are larger than donor density and when the donors and acceptors don’t satisfy Fig. 2a anymore. In this case, Fermi levels will be determined by the acceptors and band-edge “donor,” or acceptor formation energy lines and the 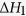 line in Fig. 5c. This is different from traditional defect analysis, which omits band-edge “defects.”

Overall, the simulated hole density at T = 300 K is smaller than 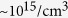 and electron density is smaller than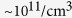 in all chemical potential regions, which agrees with experimental observations^26^. In addition, our calculation shows that the Fermi level splitting in MAPbI_3_ is 1.15 eV (see Fig. 7a), in agreement with the open circuit voltage (V_OC_) in high-efficiency MAPbI_3_ solar cells^23^. We also found that Fermi-level splitting is largest under MAI-rich conditions, in agreement with the fact that MAPbI_3_ is often grown under MAI-rich conditions. To further enhance V_OC_, p-type Fermi level can be lowered to the cross point of donor and acceptors (0.10 eV above VBM) by high-temperature growth because of two things. On one hand, band-edge “donor” 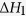 line will be higher at high temperatures. On the other hand, when the temperature is lowered rapidly after high-temperature growth, the p-type Fermi level can be further moved downward to VBM, as discussed in Ref. 32. In the case of n-type, Fermi level is also expected to move toward CBM due to quenching. Our simulation results show that if MAPbI_3_ is grown at T = 450 K, p-type Fermi level is 0.16 eV and after quenching to T = 300 K, p-type Fermi level will be 0.09 eV, about 0.1 eV smaller than the value obtained with equilibrium growth at room temperature and n-type Fermi level is 1.49 eV, which is 0.18 eV larger. As a result, the open circuit voltage can be increased by about 0.28 eV. This could explain some of the annealing approaches adopted by some experiments^33^,^34^.

## Conclusions

In conclusion, our work clarified the origin of Fermi-level pinning in different scenarios and identified the condition for self-regulation of charge-compensated defects formation in semiconductors. We proposed a new analysis method to treat band-edge thermal excitation in the same footing as defect excitation and a general criterion to judge whether thermal band-edge “defects” can be neglected and whether charge-compensated defect behavior can exist. Using NaCl and MAPbI_3_ as examples, we confirmed our concepts and explained some of the unexpected defect behaviors, such as the independence of defect formation energy with respect to the variation of atomic chemical potentials. Our work enriches the understanding of defect physics and will be very useful for future design and analysis of defect properties.

## Methods

### First-principles calculations

Our first-principles total energy and band structure calculations are performed using density functional theory (DFT)^35^,^36^ as implemented in the VASP code^37^,^38^. The electron and core interactions are included using the frozen-core projected augmented wave (PAW) approach^39^. The defect properties are calculated using the scheme described in Ref. 2. For describing the concept, only dominant vacancies are considered as our calculations show that other defects are not important in our studied systems. But our concept is also valid if dominant defects are not just vacancies,(e.g., in CuInSe_2_, the dominant donor is In_Cu_ cation anti-site)^40^,^41^.

## Additional Information

**How to cite this article**: Yang, J.-H. *et al.* Self-regulation of charged defect compensation and formation energy pinning in semiconductors. *Sci. Rep.* **5**, 16977; doi: 10.1038/srep16977 (2015).
